# Physiological responses and evaluation of cold tolerance in red prickly ash (*Zanthoxylum bungeanum* Maxim.) germplasm under low-temperature treatment

**DOI:** 10.3389/fpls.2026.1868576

**Published:** 2026-05-28

**Authors:** Yuping Wang, Yanzhi Tan, Guowei Chen, Yahan Zhang, Junlan Liu, Meng Lin, Fang Yu, Qingbo Li, Xiaofeng Pu, Hongbing Li, Qiang Li

**Affiliations:** Chongqing Key Laboratory for Germplasm Innovation for Special Aromatic Spice Plants, College of Smart Agriculture/Institute of Special Plants, Chongqing University of Arts and Sciences, Chongqing, China

**Keywords:** antioxidant enzymes, cold tolerance evaluation, conductivity, low-temperature treatment, red prickly ash

## Abstract

This study investigated how red prickly ash germplasms respond physiologically to low-temperature treatments. Sixteen red prickly ash germplasms were utilized to examine variations in physiological indices at 0, −10, −20, and −30 °C. The results showed that low-temperature treatment led to an increase in the relative conductivity (REC) of the germplasms. The semi-lethal temperatures of germplasms WCDHP, CCSJ, and HGHJ were lower than −10 °C, while those of germplasms JYH, NLJ, and CJ were higher than −7 °C. With decreasing temperature, the levels of soluble sugar (SS) and soluble protein (SP) initially declined, subsequently increased, and finally decreased, while the concentration of free proline (PRO) initially increased and then decreased. In addition, with decreasing temperature, the activity of superoxide dismutase (SOD) increased and then decreased, whereas those of peroxidase (POD) and catalase (CAT) increased, then decreased, and then increased. HGHJ, CCSJ, and SZT had the highest comprehensive membership function scores, whereas CJ, JQWC, and NLJ had the lowest comprehensive scores. Eight germplasm lines (including CCSJ, PTJ, and HGHJ) screened using principal component analysis (PCA) showed strong cold tolerance, whereas BYH, TSWC, and CJ exhibited weak cold tolerance. In summary, CCSJ and HGHJ demonstrated strong cold tolerance, whereas NLJ and CJ demonstrated weak cold tolerance.

## Graphical Abstract

**Figure:**
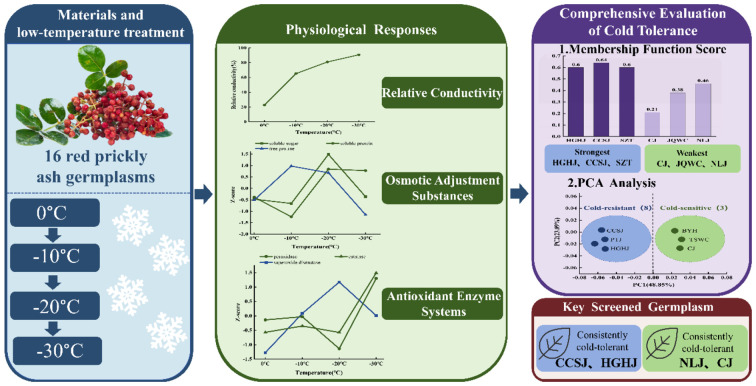

## 1 Introduction

Prickly ash is an important spice and medicinal plant that is planted worldwide owing to its unique aroma and taste (Wang et al., 2021; Liu et al., 2026). There are about 250 species of prickly ash, distributed in tropical and subtropical regions of Asia, America and Africa. China has the largest planting area and total prickly ash production in the world, particularly in southwestern and northern China. Because of their well-developed roots, prickly ash species possess drought tolerance, barren tolerance, and strong ecological adaptability and have thus gained economic importance in China for managing barren mountains, promoting ecological construction, and supporting rural revitalization (Guo et al., 2022; Tian, 2021). Two prickly ash species are found in China, red prickly ash (*Zanthoxylum bungeanum* Maxim.) and green prickly ash (*Zanthoxylum armatum* DC.) Red prickly ash originated in northern China, whereas green prickly ash originated in southwestern China (Ma, 2021). Moreover, their phenotypic characteristics, tolerance, and ecological adaptability vary due to differences in their original ecological environments (Yang et al., 2022; Dong et al., 2024a). However, only a few studies have investigated differences in cold tolerance among red prickly ash germplasms.

Temperature is the main climatic factor affecting the geographical distribution of plants and is a key factor affecting plant growth and development (Dong et al., 2024b). Under low-temperature treatments, the crop yield and quality are substantially reduced, leading to plant death (Meng et al., 2020; Wu et al., 2008). Low temperatures lead to damage and destruction of the plant cell membrane system, decreased osmotic adjustment ability, and increased levels of reactive oxygen species, resulting in irreversible damage to cells (Wang et al., 2006; Xiao et al., 2008). Cao et al. (2011) reported that under low-temperature treatment, the levels of osmotic adjustment substances and antioxidant enzyme activities in oil palm and prickly ash initially increased but then decreased with decreasing temperature (Ma et al., 2012). Sun et al. (2021) reported that the REC, protective enzyme activity, and osmotic adjustment substances in coconuts increased as the temperature decreased following low-temperature treatment. Chen et al. (2020) reported that antioxidant enzyme activities and the contents of SS, SP, and PRO in prickly ash initially decreased and then increased with decreasing temperature under low-temperature treatment, and Liu et al. (2015) observed similar changes in Juglans regia. Assessing plant cold tolerance based on individual physiological indices under low-temperature treatments may not provide an accurate or objective evaluation. Therefore, methodologies that integrate multiple physiological parameters have been developed to overcome this limitation. These approaches often employ statistical tools such as PCA, cluster analysis, and correlation analysis for a holistic assessment. Liu et al. (2010) used a logistic model to estimate the semi-lethal temperature and combined it with membership functions and correlation studies to compare cold tolerance across eight prickly ash germplasms. Similarly, Shi et al. (2023) used membership functions along with PCA and clustering techniques to evaluate the cold tolerance of 23 green prickly ash germplasms, ultimately identifying CJ and LFJ as tolerant varieties and DYSJ and HYXJ as sensitive varieties.

The evaluation of cold tolerance is the basis for plant breeding and breeding of new varieties. Many studies have reported the physiological responses of red prickly ash germplasms to low temperatures and differences in their cold tolerance. However, due to limited number of germplasms, research methods, and research areas, consensus on the physiological responses and differences in cold tolerance of red prickly ash germplasm resources under low-temperature treatments are unavailable. Therefore, this study examined 16 red prickly ash germplasms to investigate the variations in REC, osmotic adjustment substances, and antioxidant enzyme activity in response to low-temperature treatments. A comprehensive assessment of cold tolerance was conducted using membership functions, correlation analyses, and PCA. The results provide valuable theoretical insights and practical recommendations for breeding cold-tolerant red prickly ash as well as for promoting and introducing this species into colder regions.

## 2 Materials and methods

### 2.1 Overview of test site

The experiment was conducted in the prickly ash experimental base of the Chongqing University of Arts and Sciences (Yongchuan, Chongqing, China) in 2022, and the tested germplasm resources were 3-year-old prickly ash plants. The test area has a subtropical monsoon humid climate, with an average annual temperature of 17.7 °C, average annual precipitation of 1015 mm, average annual sunshine of 1218.7 h, and average annual frost-free period of 317 d. The tested site contained purple soil. The nutrient composition of 0–30 cm soil was: available nitrogen, 36.51 mg/kg; available phosphorus, 105.33 mg/kg; available potassium, 265.72 mg/kg; total nitrogen, 1.13 g/kg; total phosphorus, 15.72 g/kg; total potassium, 1.83 g/kg; and organic matter 16.21 g/kg, with a pH of 7.01.

### 2.2 Test materials

The test materials were 16 red prickly ash germplasm resources. Detailed information on each prickly ash germplasm is shown in **Table 1**.

**Table 1:** List of materials.

Number	Abbreviate	Region of origin
1	NQ1H	Hancheng, Shaanxi, China
2	NQ2H	Hancheng, Shaanxi, China
3	PTJ	Tongchuan, Shaanxi, China
4	SZT	Tongchuan, Shaanxi, China
5	RBHJ	Asakura, Japan
6	GJ	Yaan, Sichuan, China
7	NLJ	Yaan, Sichuan, China
8	TSWC	Tianshui, Gansu, China
9	SDYHJ	Zun yi, Gui zhou, China
10	CJ	Hancheng, Shaanxi, China
11	JQWC	Wudu, Gansu, China
12	CCSJ	Asakura, Japan
13	BYH	Yuncheng, Shanxi, China
14	WCDHP	Hancheng, Shaanxi, China
15	HGHJ	Hancheng, Shaanxi, China
16	JYH	Jiaozuo, Henan, China

### 2.3 Test methods

#### 2.3.1 Experimental design and sampling

In January 2022, samples were collected from the test site, and five plants with similar growth vigor were selected from each germplasm line. One-year-old branches (the xylem of the entire branch) of 25–30 cm in length and 0.8–1.0 cm in diameter were randomly cut from the middle of each tree. The branches were rinsed with pure water and sealed with paraffin to prevent water loss, then placed in the refrigerator to simulate low-temperature treatment. Four temperature treatments of 0, −10, −20, and −30 °C were used. Three branches from each prickly ash germplasm were subjected to different temperatures. The cooling rate of the freezer was set to 5 °C/h. After reaching the desired temperature, the temperature was maintained for 12 h and then raised to 5 °C for 12 h. The middle part of the branch was collected and each treatment was repeated three times to determine the physiological and biochemical indices.

#### 2.3.2 Determination of physiological and biochemical indices

REC was assessed and the semi-lethal temperature (LT50) was determined using the method described by Lu et al. (2017). The levels of SS, SP, and PRO were measured following the protocols outlined by Liu et al. (2017) and Bi et al. (2023). The activities of SOD, POD, and CAT were measured following the methods of Huang et al. (2016).

### 2.4 Data statistics and analysis

Preliminary statistics and sorting of experimental data were conducted using Excel 2019. For the statistical analysis, SPSS for Windows version 19.0 and ChiPlot mapping software were utilized for further examination. A one-way analysis of variance (ANOVA) was employed to test for significance, while Tukey’s method was used to identify statistical differences between the groups.

The Logistic equation was used to fit the treatment temperature x and the corresponding REC Y:


$$Y=\frac{k}{1+ae^{bx}}$$

where k = 100 indicates the saturation capacity of the cell injury rate. Parameters a and b and the coefficient of determination were obtained, and LT50 was calculated according to equation:


$${\text{LT}}_{50}=\frac{\text{lna}}{\text{b}}$$

The following is a comprehensive evaluation formula based on the membership function method: A positive correlation exists between the index calculated by the equation and the cold tolerance:


$$\text{f}({\text{X}}_{\text{ij}})=\frac{{\text{X}}_{\text{ij}}-{\text{X}}_{\text{jmin}}}{{\text{X}}_{\text{jmax}-\text{Xjmin}}}$$

The membership function f(xij) for variety i and index j was derived from the measured value Xij, where Xjmin and Xjmax represent the minimum and maximum values of index j, respectively. A negative correlation exists between the index calculated by the equation and the cold tolerance:


$$\text{f}({\text{X}}_{\text{ij}})=1-\frac{{\text{X}}_{\text{ij}}-{\text{X}}_{\text{jmin}}}{{\text{X}}_{\text{jmax}}-{\text{X}}_{\text{jmin}}}$$

### 2.5 Principal component analysis

The Kaiser-Meyer-Olkin (KMO) test value and Bartlett spherical test value of the data set are calculated to determine whether the data set is suitable for PCA. Subsequently, after standardizing the data, the eigenvalues of the covariance matrix were used as indicators to select the principal components, and the variance contribution rate of each extracted component was used as the weight. The weighted summation method was used to calculate the comprehensive membership function scores of various quality systems under low temperature treatment. The following formula is used for calculation:


$$\text{F}=A_{1}F_{1}+A_{2}F_{2}+A_{3}F_{3}\ldots \ldots +A_{n}F_{n}$$

where F is the comprehensive score, Fn is the score of the nth principal component, and An is the variance contribution rate of the nth principal component.

## 3 Results

### 3.1 REC and semi-lethal temperature of red prickly ash germplasms

**Figure 1:**
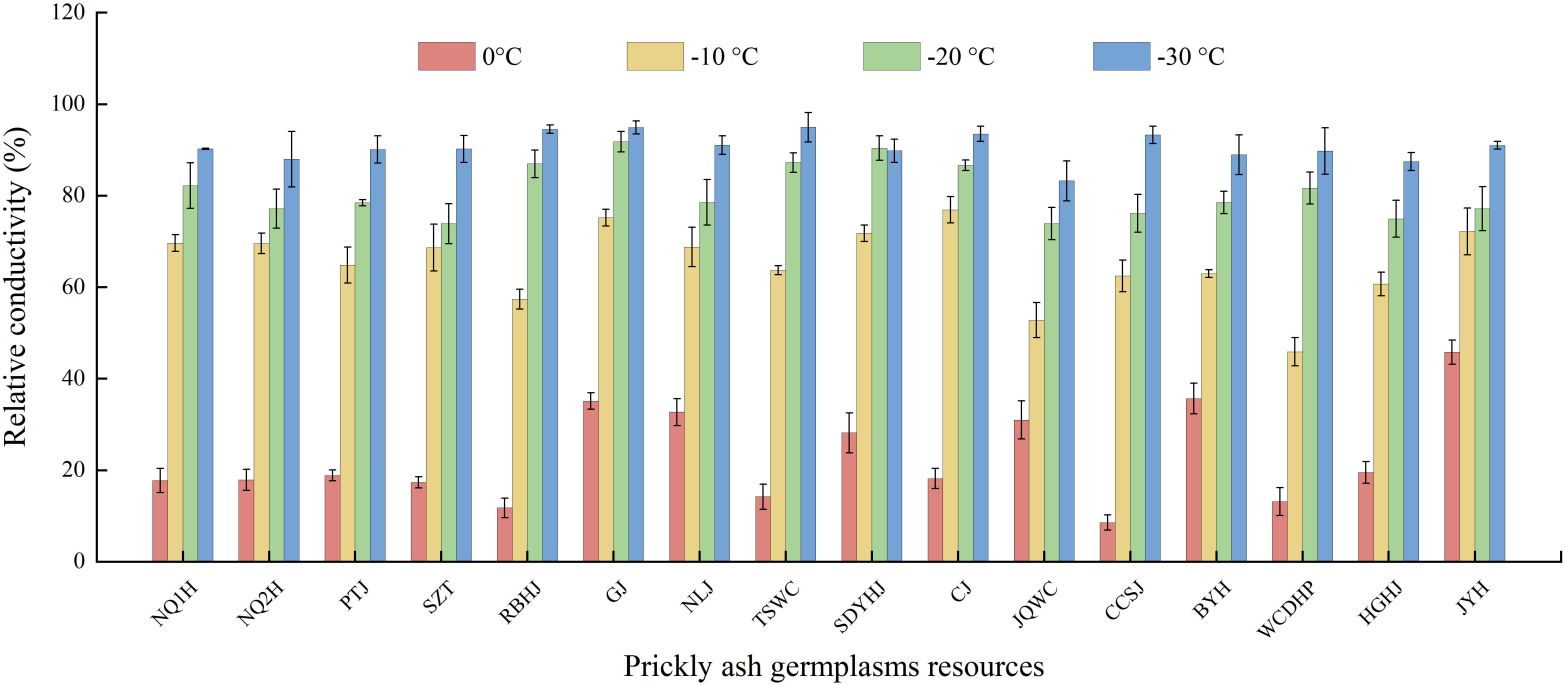
Variations in the REC of red prickly ash germplasms from diverse origins under various low-temperature treatments.

Red prickly ash germplasms exhibited significant variation in REC across different temperature treatments, with conductivity increasing markedly as the temperature decreased (**Figure 1**; **Supplementary Table 1**). In the 0 °C treatment, the electrical conductivity values of the red prickly ash germplasm ranged from 8.55–45.79%, with an average of 22.83%. Among the germplasms, the highest and lowest values were obtained for JYH and CCSJ, respectively. Under the −10 °C treatment, the electrical conductivity was 45.87–76.88%, with an average of 65.20%. Among the germplasm lines, the highest was CJ and the lowest was WCDHP. Under the −20 °C treatment, the electrical conductivity was 73.86–91.77%, with an average of 80.95%; the highest value was for GJ and the lowest was for SZT. Under the −30 °C treatment, the conductivity was 83.22–94.92%, with an average of 90.67%. Among the germplasms, TSWC and JQWC had the highest and lowest, respectively. As the temperature decreased from 0 °C to -10 °C, the REC of each red prickly ash germplasm rose by an average of 42.37%. However, when the temperature dropped further from −10 °C to −20 °C, the increase in REC slowed, with a more modest average rise of 15.75%. From −20 °C to -30 °C, the conductivity continued to increase but at a reduced rate, with the average rise being just 9.72%.

The logistic equation fitting results for the REC of the branches of each red prickly ash germplasm are shown in **Table 2**. The correlation coefficients ranged from 0.9325–0.9961, all of which were statistically significant, indicating a strong model fit. This suggests that the calculated semi-lethal temperatures were reliable and accurate. The average semi-lethal temperature of the 16 red prickly ash germplasms was −7.88 °C. Among them, WCDHP and CCSJ had the lowest semi-lethal temperatures of −12.10 °C and −11.92 °C, while JYH had the highest LT50 (-1.00 °C), respectively. Lower semi-lethal temperatures indicate stronger cold tolerance in plants, whereas higher temperatures suggest weaker tolerance. Therefore, WCDHP and CCSJ exhibited stronger cold tolerance, whereas JYH and GJ exhibited weaker cold tolerance.

**Table 2:** Logistic equation fitting results of the REC of branches of different varieties of red prickly ash under low-temperature treatment.

Variety	Logistic equation	LT50 (°C)	Degree of fitting
NQ1H	y=100/(1 + 2.8063e0.1195x)	-8.63	0.9504*
NQ2H	y=100/(1 + 2.7598e0.1094x)	-9.28	0.9325*
PTJ	y=100/(1 + 2.9751e0.1169x)	-9.33	0.9653*
SZT	y=100/(1 + 3.0708e0.1160x)	-9.67	0.9367*
RBHJ	y=100/(1 + 5.3235e0.1619x)	-10.33	0.9886*
GJ	y=100/(1 + 1.3923e0.1192x)	-2.78	0.9879*
NLJ	y=100/(1 + 1.6898e0.0963x)	-5.45	0.9773*
TSWC	y=100/(1 + 4.1739e0.1554x)	-9.20	0.9835*
SDYHJ	y=100/(1 + 1.6382e0.1064x)	-4.64	0.9644*
CJ	y=100/(1 + 2.5049e0.1316x)	-6.97	0.9465*
JQWC	y=100/(1 + 2.0812e0.0814x)	-9.01	0.9961**
CCSJ	y=100/(1 + 6.4609e0.1565x)	-11.92	0.9513*
BYH	y=100/(1 + 1.6308e0.0879x)	-5.56	0.9950**
WCDHP	y=100/(1 + 5.3298e0.1383x)	-12.10	0.9919**
HGHJ	y=100/(1 + 2.9989e0.1074x)	-10.23	0.9698*
JYH	y=100/(1 + 1.0805e0.077x)	-1.00	0.9752*

“**” indicates extremely significant difference at 1% (*P*<0.01) and “*” indicates significant difference at 5% (*P* < 0.05).

### 3.2 Effect of low temperature on osmotic adjustment substances of red prickly ash

**Figure 2:**
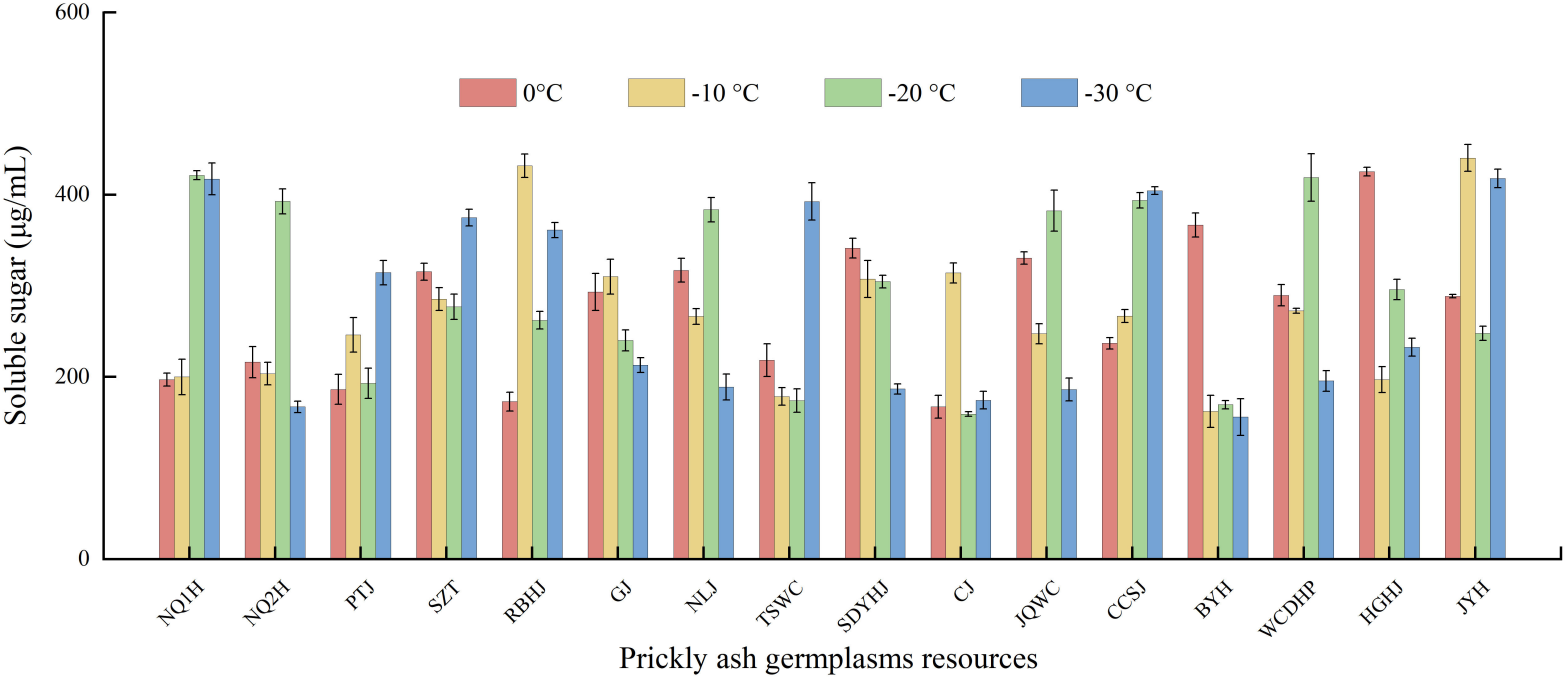
Variations in the SS content of red prickly ash germplasms from diverse origins under various low-temperature treatments.

A significant difference was observed in the SS content among the red prickly ash germplasms under different temperature treatments, and the SS content of each red prickly ash germplasm exhibited an S-shaped trend with decreasing temperature (**Figure 2**; **Supplementary Table 2**). At 0 °C, the SS content ranged from 167.23–425.47 μg/mL, with an average of 272.70 μg/mL. Among the germplasms, HGHJ had the highest sugar content, whereas CJ had the lowest. At −10 °C, SS levels ranged from 162.10–440.37 μg/mL, averaging 270.59 μg/mL. JYH had the highest value, whereas BYH had the lowest. At −20 °C, the SS content varied from 159.23–421.40 μg/mL, with an average of 294.76 μg/mL. NQ1H showed the highest content, whereas CJ showed the lowest. Under the −30 °C treatment, SS ranged from 155.93–417.80 μg/mL, with an average of 273.95 μg/mL. JYH had the highest sugar content, whereas BYH had the lowest. When the temperature decreased from 0 to -10 °C, the average SS content decreased by 0.77%. From −10 °C to −20 °C, the SS content increased by an average of 8.93%. However, from −20 °C to −30 °C, the SS content decreased by an average of 7.06%.

**Figure 3:**
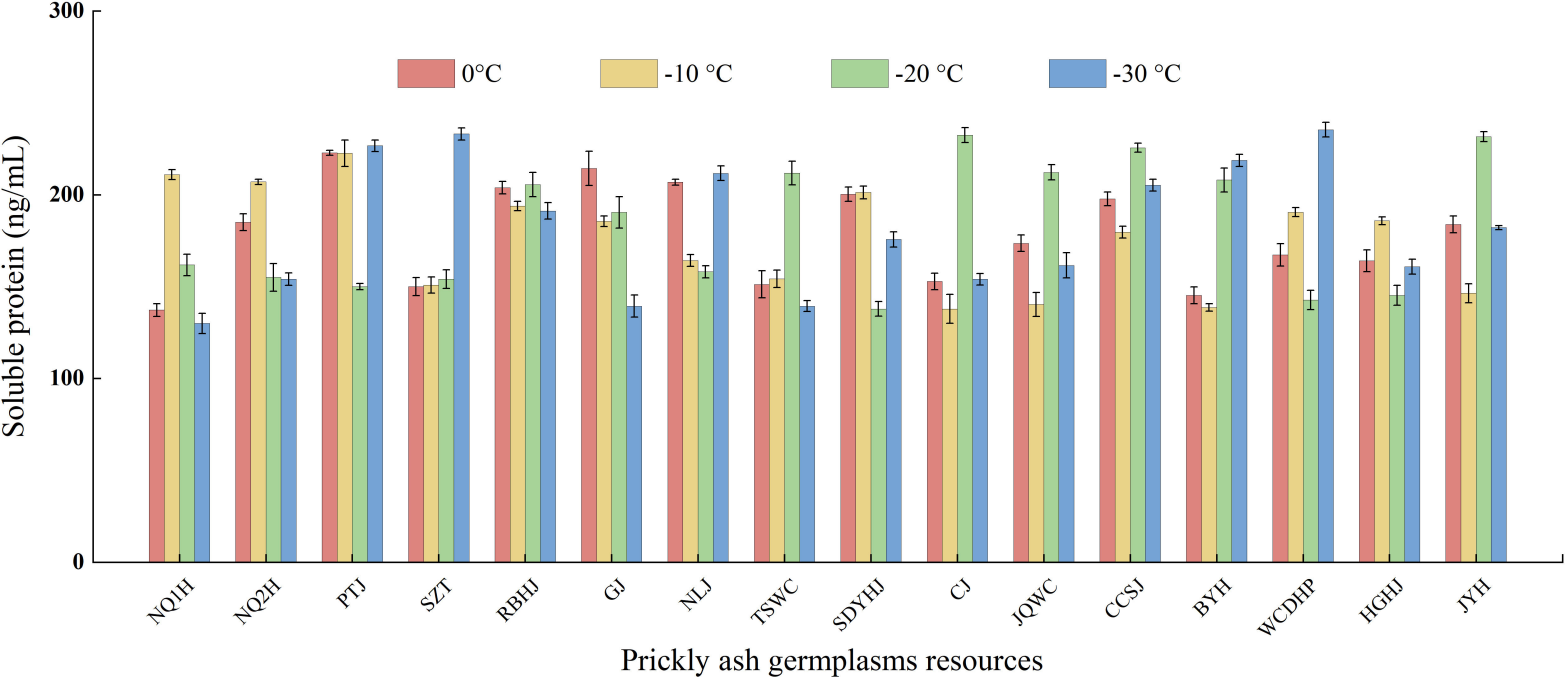
Variations in SP of red prickly ash germplasms from diverse origins under various low-temperature treatments.

The SP content of red prickly ash germplasm varied significantly across different temperature treatments, exhibiting an S-shaped trend as the temperature decreased (**Figure 3**; **Supplementary Table 3**). At 0 °C, the SP content ranged from 137.23–222.80 ng/mL, with an average value of 178.51 ng/mL. Among the germplasm lines, PTJ had the highest content, whereas NQ1H had the lowest. When the temperature was reduced to −10 °C, the SP content ranged from 137.87–222.60 ng/mL, with an average of 175.61 ng/mL. PTJ showed the highest value, whereas CJ showed the lowest. Under the −20 °C treatment, SP content ranged from 137.87–232.40 ng/mL, averaging 182.66 ng/mL. CJ had the highest content, whereas SDYHJ had the lowest. At −30 °C, the SP content varied from 129.97–235.40 ng/mL, with an average of 182.44 ng/mL. WCDHP and NQ1H exhibited the highest and lowest values, respectively. From 0 °C to −10 °C, the SP content decreased by an average of 1.63%. In contrast, between −10 °C and −20 °C, SP content increased by an average of 4.01%. However, when the temperature was further reduced from −20 °C to −30 °C, the SP content experienced a slight decrease of 0.12% on average.

**Figure 4:**
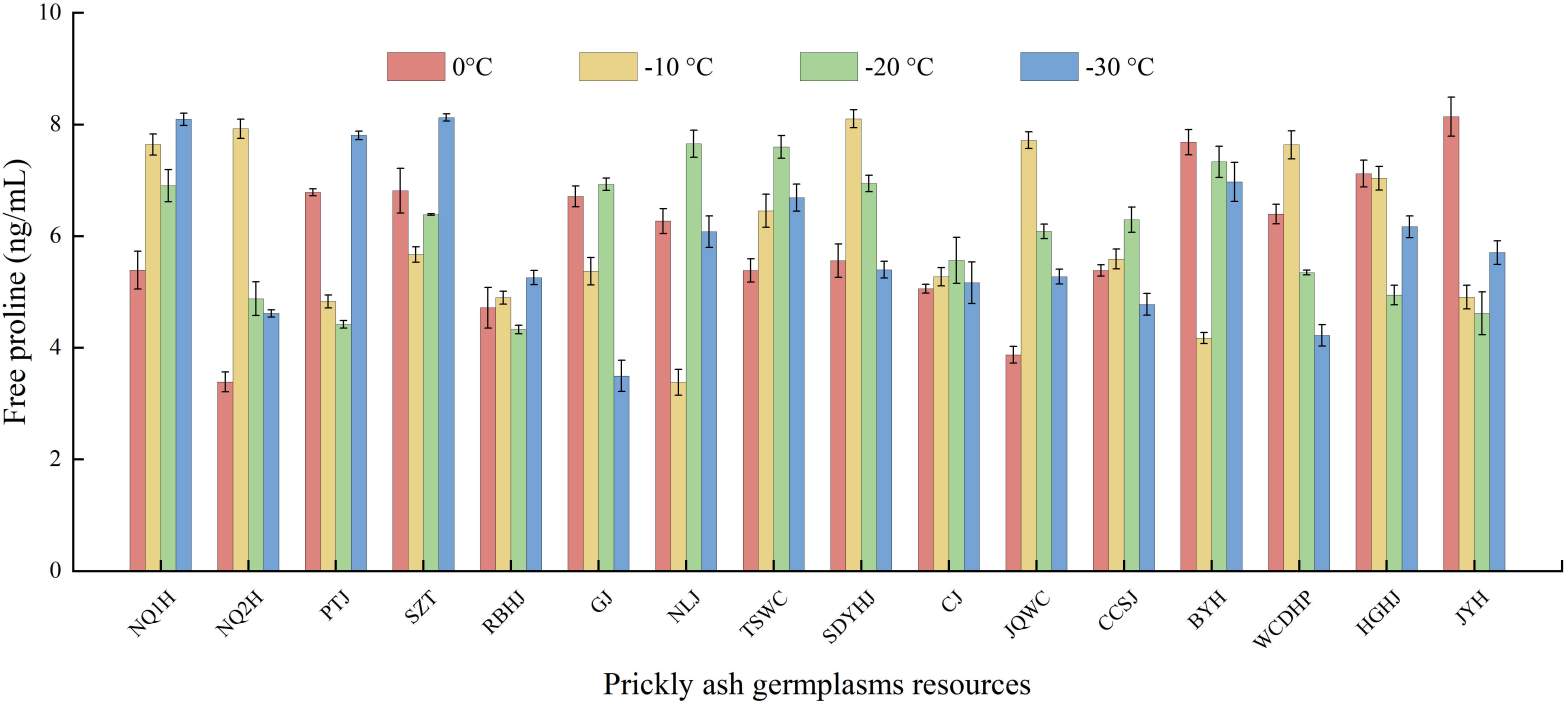
Variations in PRO of red prickly ash germplasms from diverse origins under various low-temperature treatments.

PRO content in the branches of red prickly ash germplasm varied significantly across different temperature treatments. As the temperature decreased, the PRO content initially increased, followed by a decrease (**Figure 4**; **Supplementary Table 4**). At 0 °C, the PRO levels ranged from 3.39–8.14 ng/mL, with an average value of 5.92 ng/mL. Among the germplasm lines, JYH exhibited the highest levels, whereas NQ2H had the lowest. When exposed to −10 °C, the PRO content ranged from 3.38–8.10 ng/mL, with an average of 6.04 ng/mL. SDYHJ had the highest content, whereas NLJ had the lowest. Under −20 °C conditions, the PRO content ranged from 4.33–7.66 ng/mL, with an average of 6.01 ng/mL. NLJ recorded the highest value, whereas RBHJ recorded the lowest. At −30 °C, the PRO content varied between 3.49 and 8.12 ng/mL, with an average of 5.86 ng/mL. Among the germplasm lines, SZT had the highest proline content, whereas GJ had the lowest. From 0 °C to −10 °C, the PRO content of the germplasms increased by an average of 2.04%. However, from −10 °C to −20 °C and −20 °C to −30 °C, the content decreased by averages of 0.39% and 2.28%, respectively.

### 3.3 Effects of low-temperature treatment on antioxidant enzyme systems of red prickly ash

**Figure 5:**
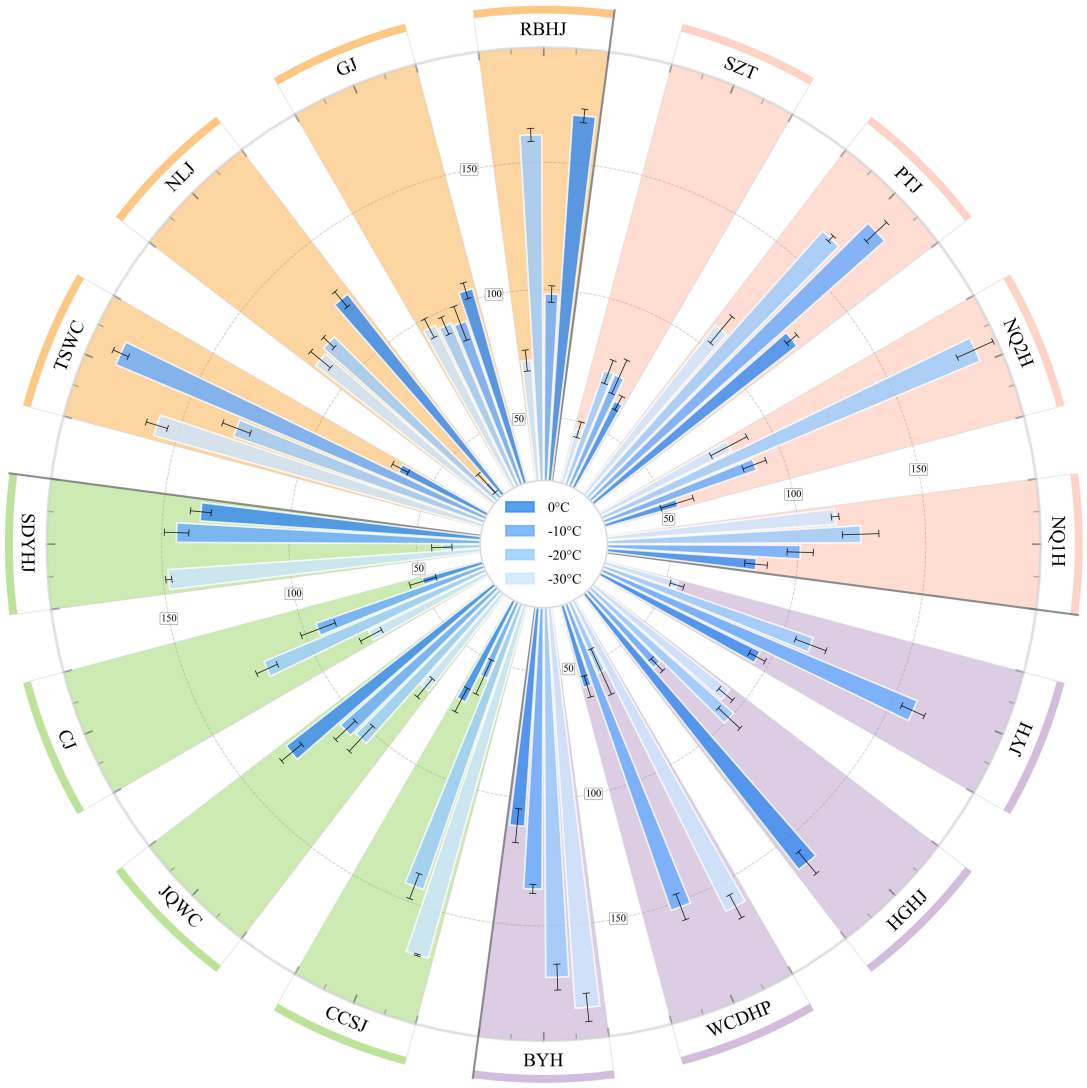
Variations in the SOD content of red prickly ash germplasms from diverse origins under various low-temperature treatments. (U/mL).

Significant variations were observed in the SOD activity of the red prickly ash germplasms under different temperature treatments. The SOD activity initially increased as the temperature decreased, followed by a subsequent decline (**Figure 5**; **Supplementary Table 5**). At 0 °C, SOD activity ranged from 49.57–168.91 U/mL, with an average of 99.88 U/mL. Among the germplasm lines, RBHJ exhibited the highest SOD activity, whereas CJ had the lowest activity. Under the −10 °C treatment, SOD activity ranged from 32.74–182.14 U/mL, with an average of 110.05 U/mL. TSWC had the highest activity, whereas NLJ had the lowest activity. At −20 °C, SOD activity varied between 39.99 and 185.80 U/mL, with an average of 118.19 U/mL. NQ2H showed the highest activity, while SDYHJ showed the lowest. For the −30 °C treatment, SOD activity ranged from 47.02–182.71 U/mL, with an average of 109.49 U/mL. At this temperature, BYH showed the highest SOD activity, whereas SZT showed the lowest. When the temperature dropped from 0 °C to −10 °C, the average SOD activity across the germplasms increased by 10.19%. From −10 °C to −20 °C, SOD activity rose by an average of 7.40%. However, from −20 °C to −30 °C, a decline in SOD activity of an average of 7.36% was observed.

**Figure 6:**
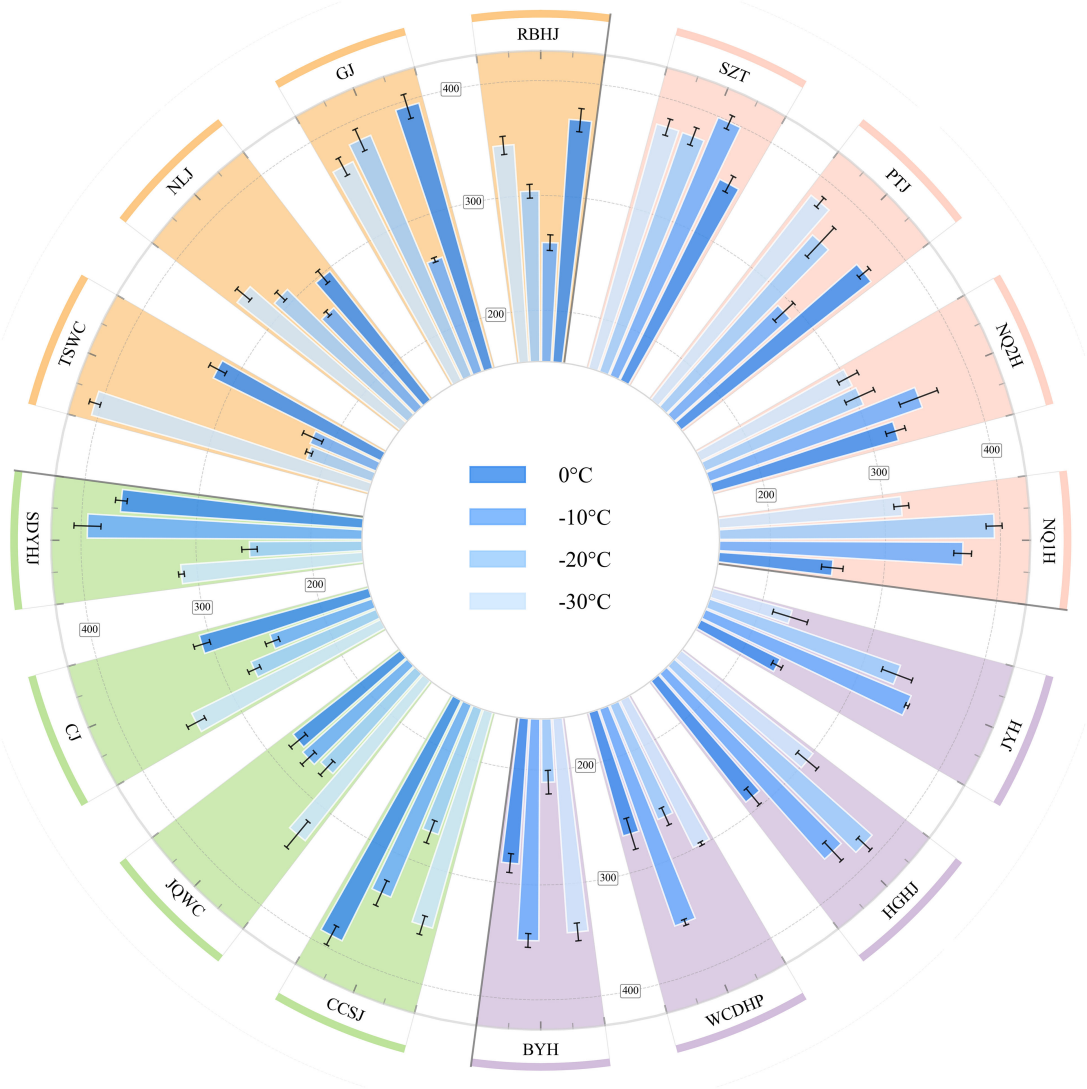
Variations in the POD content of red prickly ash germplasms from diverse origins under various low-temperature treatments. (U/mL).

Significant variations in POD activity were observed among the red prickly ash germplasms under different temperature conditions. As the temperature decreased, POD activity initially increased, then declined, and subsequently increased (**Figure 6**; **Supplementary Table 6**). At 0 °C, POD activity ranged from 232.57–394.87 U/L, with an average of 317.44 U/L. Among the germplasm lines, GJ exhibited the highest POD activity, whereas JYH exhibited the lowest. At -10 °C, POD activity ranged from 217.57–398.83 U/L, with an average of 318.65 U/L; SZT had the highest activity, and TSWC had the lowest. At -20 °C, POD activity ranged from 210.63–394.63 U/L, with an average of 306.43 U/L; NQ1H and BYH displayed the highest and lowest activity, respectively. At −30 °C, TSWC and JYH had the highest and lowest POD activities, respectively, with a range of 227.07–405.63 U/L and an average of 333.30 U/L. When the temperature dropped from 0 °C to −10 °C, the POD activity of each red prickly ash germplasm increased by an average of 0.38%. When the temperature decreased from −10 °C to −20 °C, the POD activity decreased by an average of 3.83%. Finally, when the temperature decreased from −20 °C to −30 °C, the POD activity increased by an average of 8.77%.

**Figure 7:**
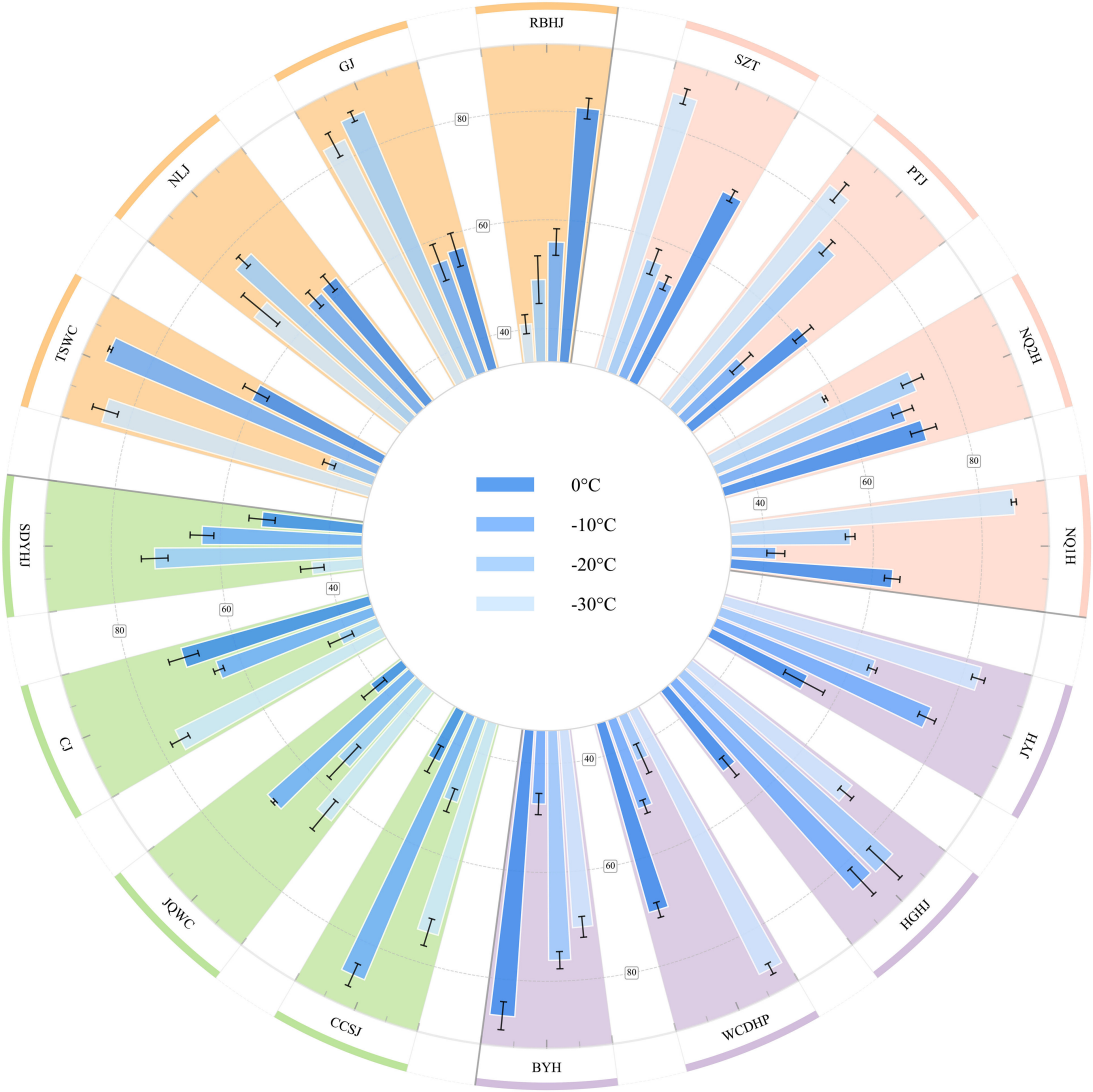
Variations in the CAT content of red prickly ash germplasms from diverse origins under various low-temperature treatments. (U/mL).

Significant variations in CAT activity were observed among the red prickly ash germplasms under the different temperature treatments. As the temperature decreased, CAT activity initially increased, then decreased, and subsequently increased (**Figure 7**; **Supplementary Table 7**). At 0 °C, CAT activity ranged from 41.10–86.72 U/mL, with an average of 62.58 U/mL. Among the germplasm lines, BYH exhibited the highest activity, whereas JQWC exhibited the lowest. At -10 °C, CAT activity ranged from 42.10–87.92 U/mL, with an average of 63.66 U/mL; TSWC had the highest activity, and NQ1H had the lowest. At −20 °C, CAT activity ranged from 41.43–86.64 U/mL, with an average of 62.57 U/mL; GJ had the highest activity, and CJ had the lowest. At −30 °C, WCDHP and RBHJ had the highest and lowest CAT activities, respectively, with a range of 40.98–87.88 U/mL and an average of 72.66 U/mL. When the temperature decreased from 0 °C to −10 °C, the CAT activity of each red prickly ash germplasm increased by 1.72%. When the temperature decreased from −10 °C to −20 °C, the CAT activity decreased by 1.72%. Finally, when the temperature decreased from −20 °C to −30 °C, the CAT activity increased by 16.13%.

### 3.4 Comprehensive evaluation of cold tolerance of red prickly ash germplasm resources

Because of the varying units and properties associated with each index, the membership function approach was employed to comprehensively evaluate the cold tolerance indices (**Table 3**). Initially, the membership function value for each index of each variety was calculated using an established formula. The average membership value for each variety was then determined. The average value increased proportionally with the cold tolerance of the plant. The average membership values for the evaluated red prickly ash germplasms ranged from 0.29–0.69, indicating significant variation in their cold tolerance capabilities. Based on the average membership values, the cold tolerance of the varieties was categorized into four distinct grades. The first grade, which represented the most cold-tolerant varieties, included PTJ and CCSJ, both of which had average membership values exceeding 0.60. The second grade, with an average membership value ranging from 0.50–0.60, included NQ1H, SZT, BYH, WCDHP, and HGHJ, indicating moderate cold tolerance. The third grade, with values between 0.40 and 0.50, was assigned to RBHJ, NLJ, TSWC, SDYHJ, NQ2H, GJ, and JYH, reflecting weaker cold tolerance. The fourth grade, indicating cold sensitivity, included CJ and JQWC with average membership values below 0.40. Among the 16 red prickly ash germplasm lines, the top five varieties in terms of average membership function values were PTJ (0.67), CCSJ (0.64), HGHJ (0.60), SZT (0.60), and WCDHP (0.52). Conversely, the five varieties with the lowest values were CJ (0.21), JQWC (0.38), NQ2H (0.43), NLJ (0.46), and GJ (0.46). As a higher average membership function value corresponds to stronger cold tolerance, the findings confirm that PTJ, CCSJ, SZT, HGHJ, and WCDHP possess superior cold tolerance, whereas CJ, JQWC, NQ2H, NLJ, and GJ exhibit weaker cold tolerance.

**Table 3:** Membership function values of cold tolerance indexes of red prickly ash.

Variety	Membership function values								Ranking
	REC	SS	SP	PRO	SOD	POD	CAT	Average	
NQ1H	0.56	0.73	0.00	1.00	0.49	0.53	0.32	0.52	7
NQ2H	0.67	0.28	0.34	0.18	0.46	0.39	0.71	0.43	14
PTJ	0.67	0.22	1.00	0.53	0.94	0.70	0.64	0.67	1
SZT	0.71	0.76	0.26	0.88	0.00	1.00	0.62	0.60	3
RBHJ	0.70	0.71	0.85	0.00	0.71	0.37	0.00	0.48	9
GJ	0.00	0.42	0.49	0.37	0.38	0.74	0.84	0.46	12
NLJ	0.39	0.59	0.55	0.47	0.38	0.20	0.64	0.46	13
TSWC	0.55	0.26	0.09	0.78	0.81	0.04	0.74	0.47	10
SDYHJ	0.25	0.56	0.41	0.77	0.62	0.52	0.07	0.46	11
CJ	0.33	0.00	0.20	0.21	0.25	0.08	0.37	0.21	16
JQWC	0.84	0.57	0.26	0.42	0.46	0.04	0.02	0.38	15
CCSJ	0.85	0.84	0.92	0.32	0.54	0.57	0.42	0.64	2
BYH	0.46	0.07	0.39	0.79	1.00	0.12	0.81	0.52	6
WCDHP	1.00	0.62	0.53	0.50	0.50	0.12	0.37	0.52	5
HGHJ	0.82	0.58	0.09	0.69	0.48	0.58	1.00	0.60	4
JYH	0.16	1.00	0.57	0.47	0.48	0.00	0.75	0.49	8

### 3.5 PCA of physiological and biochemical parameters of different varieties of red prickly ash

Using the correlation analysis method of mathematical statistics, the eigenvector (weight coefficient) of the three principal components (w1, w2, and w3) was obtained by calculating the eigenvalue and factor load matrix of each index (**Table 4**).

**Table 4:** Weighted coefficients of physiological indices across the three main components.

Physiological index	Weight coefficient of each index under each principal component		
	w1	w2	w3
REC	0.04	0.09	-0.54
SS	0.07	0.69	-0.40
SP	0.96	0.10	0.00
PRO	-0.70	0.16	0.29
SOD	0.35	-0.65	0.19
POD	0.12	0.77	0.37
CAT	-0.16	0.02	0.81

PCA was conducted on the physiological and biochemical parameters associated with cold tolerance in the 16 red prickly ash germplasms. The analysis revealed that the first three principal components accounted for 63.97% of the total variance (**Table 5**). As a result, the seven physiological indices were reduced to three composite indices that were used to evaluate the cold tolerance of red prickly ash varieties.

**Table 5:** Main component analysis of the physiological indexes.

Ingredient	Principal component eigenvalues		
	Characteristic value λ	Proportion of factors %	Total percentage%
1	1.76	25.13	25.13
2	1.59	22.71	47.84
3	1.13	16.14	63.97

Data processing and standardization were performed using SPSS and Microsoft Excel. The standardized values of the comprehensive indices for each principal component obtained through the PCA were multiplied by their respective index coefficients to calculate the scores for the various factors (F1, F2, and F3). The weights of each of these composite indices were determined, yielding values of 0.393, 0.355, and 0.252 for the three principal components. Using these weights, the comprehensive score (F) for each germplasm was calculated to represent the cold tolerance. The cold tolerance rankings of the varieties were established based on the F values, with higher F values indicating stronger cold tolerance (**Table 6**).

**Table 6:** Comprehensive evaluation results of cold tolerance of 16 red prickly ash germplasm resources.

Name	Scores in F1	Scores in F2	Scores in F3	Total scores	Total rank
NQ1H	-0.13	0.09	0.62	0.14	12
NQ2H	0.08	-0.08	0.68	0.18	9
PTJ	0.26	-0.19	0.80	0.23	3
SZT	-0.15	0.37	0.66	0.24	2
RBHJ	0.32	-0.06	0.50	0.23	4
GJ	0.07	0.07	0.65	0.22	5
NLJ	0.06	0.02	0.59	0.18	8
TSWC	-0.04	-0.26	0.74	0.08	16
SDYHJ	0.04	0.00	0.55	0.15	11
CJ	0.00	-0.14	0.58	0.10	14
JQWC	0.04	-0.06	0.52	0.12	13
CCSJ	0.22	0.10	0.60	0.27	1
BYH	0.06	-0.36	0.80	0.10	15
WCDHP	0.07	-0.04	0.62	0.17	10
HGHJ	-0.08	0.05	0.77	0.18	7
JYH	0.09	0.07	0.50	0.19	6

Principal Component 1 included eight varieties: CCSJ, HGHJ, and SZT. This component had a characteristic value of 1.76, contributing 25.13% to the total variance, and was primarily associated with SP and PRO. The varieties in this component exhibited high F values, indicating strong cold tolerance. Principal Component 2 included five varieties (NQ2H, NLJ, and JQWC), with a characteristic value of 1.59 and a contribution rate of 22.71%. This component was mainly influenced by SS, POD, and SOD, with moderate F values, suggesting an average cold tolerance. Finally, Principal Component 3 covered three varieties, including BYH, TSWC, and CJ, with a characteristic value of 1.13 and a contribution rate of 16.14%. This component was largely associated with REC and CAT, and low F values indicated relatively weak cold tolerance in these varieties.

### 3.6 Correlation analysis between semi-lethal temperature and physiological indices of different varieties of red prickly ash

**Figure 8:**
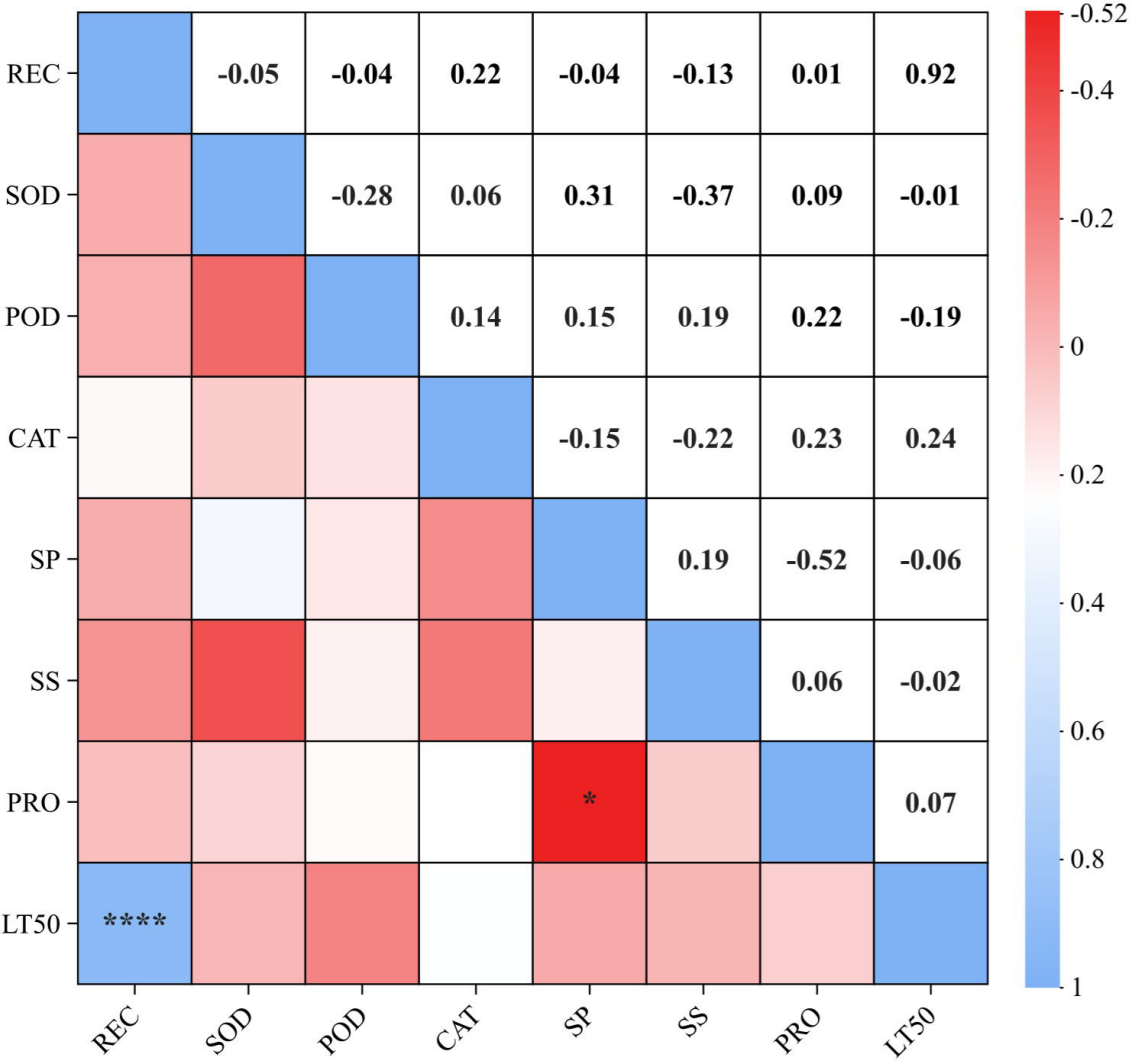
Correlation analysis of cold tolerance indices and their membership function values in red prickly ash. * Indicates a significant correlation at the 5% level (*p* < 0.05); **** Indicates an extremely significant correlation at the 0.01% level (*p* < 0.0001).

The relationships between LT50 and various physiological indices of the 16 red prickly ash germplasms were examined, revealing significant correlations among the indices (**Figure 8**). Specifically, LT50 was positively correlated with REC, CAT, and PRO activity, with correlation coefficients of 0.89, 0.43, and 0.46, respectively. Additionally, REC was positively correlated with PRO and CAT activity, with correlation coefficients of 0.47 and 0.54, respectively. A negative correlation was observed between REC and POD activity, with a coefficient of -0.42. Further analysis of LT50, along with other physiological indicators such as REC, CAT, POD, SOD, SP, PRO, and SS, revealed moderate correlations. However, the overlap among these correlations, along with the relatively low correlation coefficients, indicates that relying on a single physiological index is insufficient to accurately assess the cold tolerance of red prickly ash.

## 4 Discussion

### 4.1 Physiological response of red prickly ash to low-temperature treatment

Low-temperature treatment can lead to reduced semi-permeability of plant cell membranes and increased electrical conductivity, which negatively affects plant cold tolerance. Consequently, the semi-lethal temperature derived by fitting a logistic equation to electrical conductivity serves as a key physiological index for assessing plant cold tolerance (Wang et al., 2026). Liu (2005) found that the semi-lethal temperatures of three red prickly ash were between -20.20 °C and -17.07 °C, while the semi-lethal temperatures of the red prickly ash germplasms in the present study were between -12.1 °C and -1 °C. The difference between the two findings may have been caused by differences in climatic conditions in the introduction area (Yao et al., 2023). When a source of Wudu red prickly ash was planted in the cold northwest region and allowed long-term cold acclimation, its semi-lethal temperature reached -19.95 °C; when the same source was introduced to the warm southwest region, its cold tolerance decreased significantly, with a semi-lethal temperature of -9.01 °C (Zheng et al., 2022). Owing to differences in environmental factors such as latitude, altitude, and annual precipitation between the north and south, the cold tolerance of the same variety of red prickly ash was significantly different between the two regions (Yao et al., 2023; Xu, 2020). Further analysis indicated that as the temperature dropped from 0 °C to -10 °C, the REC of red prickly ash rose quickly, while from -10 °C to -30 °C, the increase was much slower. This suggests that the cell membrane suffered significant damage at -10 °C. The semi-lethal temperature of -12.1 °C to -1 °C observed in our study corroborated this result. The semi-lethal temperatures of the 16 red prickly ash germplasms ranged from >10 °C to <-10 °C, but only four had semi-lethal temperatures lower than -10 °C, indicating that there were significant differences in cold tolerance among the germplasms. The red prickly ash germplasms that were observed to have strong cold tolerance were consistent with those found in previous studies.

Osmotic regulatory substances are plant defense mechanisms against external damage that can effectively prevent the loss of plant water, increase cell-fluid concentrations, and enhance hydrostatic control (Bai et al., 2023; Yang et al., 2020). In the early stages of low-temperature treatment, the proline content in olive and walnut leaves increased sharply before gradually decreasing (Ling et al., 2015; Wang et al., 2019). Similarly, we found that the PRO content in red prickly ash branches increased rapidly during the initial low-temperature treatment phase, reducing cell water potential, enhancing cell stability, and improving water retention. At -10 °C, the PRO content of each red prickly ash germplasm reached its peak; the proline content continued to decrease with further reduction in temperature, indicating that the branches could not be physiologically regulated at this temperature, consistent with previous studies. The levels of SS and proteins in red prickly ash branches initially decreased slightly, likely due to the failure of the plant to adapt to the low-temperature treatment. Subsequently, both SS and proteins increased significantly, increasing the cell fluid concentration, enhancing water retention, and low-temperature adaptability. However, as the temperature decreases beyond the tolerance limits of the cells, damage to the cell membrane structure occurs, leading to an increase in hydrolytic enzyme activity. The synthesis of soluble sugar and proteins was blocked and the contents of both were significantly reduced, which is consistent with the results of previous studies (Xie et al., 2023; Li et al., 2017).

Under stress, large amounts of reactive oxygen species accumulate in plant cells, resulting in toxic effects on normal physiological functions (Xie et al., 2023). Antioxidant enzyme systems (CAT, SOD, and POD) are the primary active oxygen scavengers in plants, helping to reduce cell damage from reactive oxygen species and maintain normal cellular physiological functions (Xie et al., 2023). Liu et al. (2021) observed that SOD and POD activities in prickly ash initially decreased, then increased, and then decreased again as the temperature decreased. In contrast, this study found that in red prickly ash, SOD activity initially increased and then decreased with decreasing temperature, whereas POD and CAT activities increased, decreased, and then increased again, which was not consistent with previous results. This is mainly because the predecessor set a higher treatment temperature, which is not beyond the scope of the low-temperature treatment regulation. In the early stages of low-temperature treatment, the activities of the three enzymes increased, which alleviated the degree of damage caused by low temperatures. This demonstrates that SOD, POD, and CAT can protect plants from low-temperature environments (Li et al., 2021). With the aggravation of treatment, the accumulation of reactive oxygen species in plants exceeds the regulatory range of plants, resulting in a decrease in the activities of the three enzymes to varying degrees. The activities of POD and CAT increased at -30 °C. From 0 °C to -20 °C, the activity of SOD in the cells may have increased significantly, which catalyzed the conversion of superoxide anion into H_2_O_2_ and O_2_, and the accumulation of H_2_O_2_ stimulated the activities of POD and CAT to a certain extent, improving the scavenging ability of their defense system. However, once the temperature exceeds the lower limit of plant tolerance, the protective enzyme system is damaged and the activities of POD and CAT are reduced. This is consistent with the results obtained for Michelia plants (Chen et al., 2016) and grapes (Luo et al., 2018). The research results can provide valuable theoretical basis and practical suggestions for the cultivation of cold-tolerant red prickly ash germplasm resources for promotion and introduction in cold regions.

### 4.2 Comprehensive evaluation of cold tolerance of red prickly ash germplasm resources

Cold tolerance in plants is a complex physiological and biochemical process affected by multiple factors. It is difficult to comprehensively and objectively determine the degree of cold tolerance in plants using a single index or method (Wei et al., 2023). Liu et al. (2017) used a combination of semi-lethal temperatures, osmotic adjustment substances, and antioxidant enzyme activities to comprehensively evaluate the membership functions of prickly ash in eight regions. The cold tolerance of prickly ash in Shexian County was the strongest, whereas that in Hanyuan County was the weakest. In the present study, semi-lethal temperatures, membership function method, and PCA were used to comprehensively evaluate the cold tolerance of 16 red prickly ash germplasms. The semi-lethal temperatures of HGHJ, CCSJ, and WCDHP were all below -10 °C, indicating strong cold tolerance, while those of NLJ, CJ, and JYH were above -7 °C, indicating weak cold tolerance. Among the comprehensive membership function scores, HGHJ, CCSJ, and SZT were the highest, indicating the strongest cold tolerance, while CJ, JQWC, and NLJ exhibited the lowest cold tolerance. PCA divided the 16 red prickly ash germplasm lines into three groups. The first group comprised eight germplasms, including CCSJ, PTJ, and HGHJ, with a contribution rate of 25.13% and strong cold tolerance. The second group included five germplasms, NQ2H, NLJ, and JYH, with a contribution rate of 22.71% and normal cold tolerance. The third group included three germplasms, BYH, TSWC, and CJ, with a contribution rate of 16.14% and the weakest cold tolerance. In summary, CCSJ and HGHJ demonstrated strong cold tolerance in the three evaluation methods and were considered cold-tolerant varieties, whereas NLJ and CJ exhibited poor cold tolerance in the three evaluation methods and were considered cold-sensitive varieties.

## 5 Conclusions

Under low-temperature treatment, the physiological responses of red prickly ash germplasms were significantly different. With decreasing temperature, the REC of the red prickly ash germplasm increased rapidly, the treatment intensified, and the rate of increase gradually decreased. The semi-lethal temperatures of the red prickly ash germplasms ranged from -12.10 °C to -1.00 °C. The SS and protein contents first decreased, then increased, and then decreased with decreasing temperature, whereas the PRO content first increased and then decreased. An increase in the early stage of low-temperature treatment can increase the concentration of cell sap, increase the water-holding capacity of the cells, and improve the adaptability to low temperatures. When the treatment intensifies beyond the range of cell tolerance, the cell membrane structure is damaged. The activity of SOD first increased and then decreased with decreasing temperature, whereas the activities of POD and CAT first increased, then decreased, and then increased. The activity of antioxidant enzymes increases during the initial stages of low-temperature treatment, which improves the ability of plants to resist low temperatures. With the aggravation of treatment, the accumulation of reactive oxygen species in plants exceeds their regulatory range and the activity of antioxidant enzymes is reduced to varying degrees. Using semi-lethal temperatures and the membership function method, PCA, CCSJ, and HGHJ were identified as having strong cold tolerance and were considered cold-tolerant varieties, whereas NLJ and CJ demonstrated poor cold tolerance in the three evaluation methods and were considered sensitive to cold.

## Data Availability

The original contributions presented in the study are included in the article/**Supplementary Material**. Further inquiries can be directed to the corresponding author.
