# Supplementary material for: Physiological responses and evaluation of cold tolerance in red prickly ash (Zanthoxylum bungeanum Maxim.) germplasm under low-temperature treatment
Source: Front Plant Sci. 2026 May 28;17:1868576. doi: 10.3389/fpls.2026.1868576 (PMC13253291; doi:10.3389/fpls.2026.1868576)
Supplement: Supplementary file 1 [file DataSheet1.pdf]

Supplementary Material

1 Supplementary Tables

Table S1. Changes of REC content in red prickly ash germplasm from different origins under different low temperature treatment.

Variety	REC (%)			
	0 °C	−10 °C	−20 °C	−30 °C
NQ1H	17.76±2.64de	69.63±1.83abc	82.18± 4.97abcde	90.19±0.13ab
NQ2H	17.84±2.30de	69.53±2.24abc	77.12±4.26cde	87.96±6.05ab
PTJ	18.84±1.18de	64.83±3.92bcd	78.42±0.67cde	90.10±2.99ab
SZT	17.30±1.21de	68.61±5.11abc	73.86±4.39e	90.18±2.95ab
RBHJ	11.75±2.12ef	57.38±2.17de	86.95±3.01abc	94.51±0.89a
GJ	35.10±1.81b	75.17±1.83a	91.77±2.21a	94.90±1.42a
NLJ	32.68±2.93b	68.77±4.28abc	78.52±5.00cde	91.05±2.03ab
TSWC	14.19±2.75ef	63.67±0.98bcd	87.22±2.14abc	94.92±3.20a
SDYHJ	28.14±4.35bc	71.78±1.80ab	90.38±2.66ab	89.79±2.54ab
CJ	18.17±2.21de	76.88±2.89a	86.62±1.12abcd	93.47±1.66a
JQWC	30.97±4.14b	52.78±3.80ef	73.88±3.52e	83.22±4.36b
CCSJ	8.55±1.69f	62.46±3.46cd	76.13±4.14de	93.28±1.90a
BYH	35.64±3.36b	62.94±0.87bcd	78.51±2.44cde	88.94±4.34ab
WCDHP	13.13±3.07ef	45.87±3.06f	81.64±3.50abcde	89.73±5.09ab
HGHJ	19.50±2.36de	60.67±2.57cde	74.91±4.03e	87.45±1.98ab
JYH	45.79±2.63a	72.18±5.11ab	77.14±4.84cde	90.98±0.85ab
Average value	22.83±1.16cd	65.20±0.68bcd	80.95±1.04bcde	90.67±0.60ab
F value	45.02**	20.44**	8.21**	3.01**

Note: Different lowercase letters in the same column represent significant difference ($P<0.05$), "**" means the difference was extremely significant at 1% level ($P<0.01$), "*" means the difference was significant at 5% level ($P < 0.05$), the same below.

Table S2. Changes of SS content in red prickly ash germplasm from different origins under different low temperature treatment.

Variety	SS (μg/mL)			
	0 °C	−10 °C	−20 °C	−30 °C
NQ1H	197.17±7.08hi	200.00±19.45f	421.40±4.92a	417.27±17.47a
NQ2H	216.27±17.07gh	203.60±12.34f	392.73±13.65ab	167.13±6.18hi
PTJ	186.30±16.40hi	246.13±19.00e	193.00±16.59f	314.43±13.41d
SZT	315.50±9.34cd	285.53±12.48bcde	277.00±13.79cde	374.80±9.20bc
RBHJ	172.90±10.33i	431.63±12.90a	262.13±9.62de	361.20±8.20c

GJ	293.30±20.33de	310.00±19.25bc	240.13±11.55e	213.07±8.15fg
NLJ	317.03±12.98cd	266.33±8.60de	383.50±13.34ab	188.87±14.32ghi
TSWC	218.37±17.81gh	178.53±9.62f	174.03±12.81f	392.60±20.36abc
SDYHJ	341.33±10.88bc	307.40±20.36bcd	304.63±6.91c	186.80±5.57ghi
CJ	167.23±12.53i	314.07±11.12b	159.23±2.66f	174.47±9.62hi
JQWC	330.47±6.74bc	247.33±10.95e	382.47±22.41b	186.27±12.60ghi
CCSJ	236.97±6.27fg	266.87±7.16de	393.73±8.49ab	404.43±4.12ab
BYH	366.63±13.17b	162.10±17.52f	169.47±4.45f	155.93±20.05i
WCDHP	289.67±11.61de	272.53±2.66bcde	418.83±26.15ab	195.53±11.38gh
HGHJ	425.47±4.74a	197.03±14.15f	295.93±11.07cd	232.60±9.91f
JYH	288.63±1.79de	440.37±14.71a	247.93±7.85e	417.80±10.25a
Average value	272.70±5.07ef	270.59±0.73cde	294.76±3.37cd	273.95±1.94e
F value	113.96**	95.96**	151.86**	214.04**

Table S3. Changes of SP content in red prickly ash germplasm from different origins under different low temperature treatment.

**Variety**	**SP (ng/mL)**			
	**0 °C**	**−10 °C**	**−20 °C**	**−30 °C**
NQ1H	137.23±3.42j	210.97±2.63ab	161.80±5.80f	129.97±5.45h
NQ2H	185.07±4.54de	207.00±1.41b	155.00±7.46fg	154.07±3.35g
PTJ	222.80±1.41a	222.60±7.20a	149.97±1.72fgh	226.63±3.16ab
SZT	149.97±4.87hij	150.90±4.40ij	154.10±5.03fg	233.10±3.29a
RBHJ	203.80±3.40bc	193.87±2.60cd	205.57±6.60cd	191.20±4.50e
GJ	214.30±9.30ab	185.63±2.87def	190.47±8.56de	139.40±5.99h
NLJ	206.83±1.67bc	164.17±3.20gh	158.07±3.31fg	211.70±4.00cd
TSWC	151.27±7.33hij	154.23±4.78hi	211.83±6.41bc	139.40±3.00h
SDYHJ	200.27±3.88bc	201.23±3.54bc	137.87±3.97h	175.67±4.11f
CJ	152.80±4.55ghi	137.87±7.93k	232.40±4.06a	154.03±3.15g
JQWC	173.60±4.51ef	140.33±6.52jk	212.20±4.16bc	161.60±6.92g
CCSJ	197.80±3.73cd	179.63±3.20ef	225.60±2.44ab	205.23±3.20d
BYH	145.27±4.59ij	138.63±2.01jk	208.03±6.52c	218.70±3.35bc
WCDHP	167.23±6.07fg	190.50±2.44cde	142.77±5.26gh	235.40±4.01a
HGHJ	164.03±5.87fgh	185.83±2.16def	145.23±5.46gh	160.87±4.05g
JYH	183.93±4.57de	146.37±5.12ijk	231.60±2.65a	182.10±1.10ef
Average value	178.51±1.24ef	175.61±1.21fg	182.66±0.46e	182.44±0.72ef
F value	88.14**	127.72**	130.26**	222.88**

Table S4. Changes of PRO content in red prickly ash germplasm from different origins under different low temperature treatment.

**Variety**	**PRO (ng/mL)**			
	**0 °C**	**−10 °C**	**−20 °C**	**−30 °C**

NQ1H	5.39±0.34ghi	7.64±0.19a	6.90±0.29cd	8.09±0.11a
NQ2H	3.39±0.18j	7.92±0.17a	4.88±0.30ij	4.61±0.07hi
PTJ	6.79±0.06cd	4.83±0.11f	4.42±0.07j	7.80±0.07a
SZT	6.81±0.40cd	5.67±0.14de	6.38±0.01de	8.12±0.07a
RBHJ	4.72±0.36i	4.90±0.11f	4.33±0.08j	5.26±0.13efg
GJ	6.71±0.19cd	5.37±0.25ef	6.93±0.11cd	3.49±0.28j
NLJ	6.27±0.22def	3.38±0.23h	7.66±0.24a	6.08±0.28cd
TSWC	5.38±0.21ghi	6.45±0.30c	7.60±0.20ab	6.69±0.24bc
SDYHJ	5.56±0.30fgh	8.10±0.16a	6.94±0.15bcd	5.40±0.15efg
CJ	5.06±0.08hi	5.27±0.16ef	5.56±0.41fgh	5.17±0.37fgh
JQWC	3.87±0.15j	7.72±0.15a	6.08±0.13ef	5.27±0.13efg
CCSJ	5.38±0.10ghi	5.59±0.18de	6.29±0.22de	4.78±0.20ghi
BYH	7.68±0.23ab	4.17±0.10g	7.33±0.28abc	6.97±0.35b
WCDHP	6.39±0.17de	7.64±0.25a	5.35±0.04ghi	4.22±0.19i
HGHJ	7.12±0.24bc	7.04±0.21b	4.95±0.18hij	6.17±0.19cd
JYH	8.14±0.35a	4.91±0.21f	4.61±0.38j	5.70±0.21def
Average value	5.92±0.03efg	6.04±0.05cd	6.01±0.04efg	5.86±0.09de
F value	85.47**	179.57**	78.60**	124.65**

Table S5. Changes of SOD content in red prickly ash germplasm from different origins under different low temperature treatment.

Variety	SOD (U/mL)			
	**0 °C**	**−10 °C**	**−20 °C**	**−30 °C**
NQ1H	83.70±4.49e	100.78±5.13def	124.48±7.17de	114.90±1.46d
NQ2H	54.78±6.65gh	88.45±4.81f	185.80±7.75a	82.22±7.97fg
PTJ	126.31±2.61b	179.15±5.46a	164.63±1.44b	109.30±6.72d
SZT	62.68±2.97fgh	72.08±7.22g	72.30±4.81i	47.02±3.19h
RBHJ	168.91±2.65a	98.28±3.22def	160.81±2.59b	72.44±4.17g
GJ	104.14±3.22cd	92.69±7.07ef	94.09±3.64h	95.96±4.11e
NLJ	124.77±3.85b	32.74±4.92h	115.69±2.87defg	113.70±5.06d
TSWC	63.65±3.52fg	182.14±3.22a	128.88±5.82cd	158.81±4.34bc
SDYHJ	135.18±4.06b	144.17±4.81bc	39.99±4.02j	147.82±1.20c
CJ	49.57±5.37h	94.62±7.14def	119.13±4.48def	76.68±4.71g
JQWC	128.43±5.14b	106.77±5.34de	104.79±6.76fgh	73.21±4.67g
CCSJ	69.24±5.22f	57.22±7.38g	143.60±5.20c	168.84±0.33b
BYH	111.07±6.67c	135.49±1.77c	170.17±5.10ab	182.71±5.56a
WCDHP	58.44±4.37fgh	152.26±5.34b	54.90±9.75j	160.93±5.04bc
HGHJ	162.36±5.50a	64.93±3.29g	99.82±5.80gh	92.68±3.52ef
JYH	94.81±3.53de	159.01±4.94b	112.00±6.39efg	54.70±2.73h
Average value	99.88±1.56cd	110.05±1.29d	118.19±0.84def	109.49±0.33d
F value	214.70**	207.68**	160.75**	271.14**

Table S6. Changes of POD content in red prickly ash germplasm from different origins under different low temperature treatment.

Variety	POD (U/L)			
	0 °C	−10 °C	−20 °C	−30°C
NQ1H	254.77±9.47ij	367.23±7.76c	394.63±6.96a	315.10±6.58fg
NQ2H	323.07±8.65d	351.43±17.51cd	304.53±13.61d	302.50±9.50g
PTJ	364.97±5.89bc	290.30±11.30f	355.67±17.25bc	381.37±5.13ab
SZT	350.53±7.71c	398.83±6.31a	375.50±7.95ab	375.90±7.38bc
RBHJ	367.03±10.13bc	259.03±6.83gh	303.83±6.00d	344.97±7.93de
GJ	394.87±10.90a	260.73±2.05gh	382.10±10.27ab	367.70±8.48bcd
NLJ	297.33±6.87efg	270.67±2.62fgh	310.10±5.28d	335.50±7.69ef
TSWC	317.93±7.84de	217.57±9.25i	215.47±2.80f	405.63±4.99a
SDYHJ	366.37±5.11bc	394.40±11.83ab	253.40±6.81e	313.83±2.52fg
CJ	308.30±7.30def	249.40±6.26h	273.23±5.71e	338.90±8.64ef
JQWC	273.63±8.76ghi	277.20±7.75fg	271.83±8.26e	332.13±14.70ef
CCSJ	389.03±9.25ab	336.83±11.88de	271.13±10.30e	350.40±8.29cde
BYH	282.20±8.20gh	348.47±6.01cd	210.63±10.30f	342.30±7.79de
WCDHP	267.10±14.03hi	355.33±2.70cd	263.50±8.16e	298.57±1.58g
HGHJ	289.40±8.98fgh	371.43±10.90bc	385.93±7.58a	300.93±11.18g
JYH	232.57±4.74j	349.50±1.57cd	331.40±13.96cd	227.07±15.78h
Average value	317.44±1.83de	318.65±1.18e	306.43±1.66d	333.30±1.38ef
F value	99.43**	126.69**	116.99**	67.68**

Table S7. Changes of CAT content in red prickly ash germplasm from different origins under different low temperature treatment.

Variety	CAT (U/mL)			
	0 °C	−10 °C	−20 °C	−30 °C
NQ1H	63.73±1.43cde	42.10±1.65h	55.77±0.88de	86.18±0.47a
NQ2H	72.44±2.43b	69.88±2.00c	73.67±2.06b	57.84±0.31e
PTJ	61.12±2.06e	49.07±2.44g	75.23±1.58b	84.37±2.05a
SZT	72.80±1.12b	53.05±1.34fg	55.87±2.46de	86.45±1.44a
RBHJ	80.79±1.91a	55.93±2.44f	49.01±4.39ef	40.98±1.76f
GJ	57.00±3.18efg	55.75±3.54f	86.64±0.99a	83.46±2.44a
NLJ	62.74±1.76cde	62.26±1.84e	76.42±1.30b	68.22±4.20cd
TSWC	60.40±2.50ef	87.92±0.31a	42.71±1.14fg	84.82±2.43a
SDYHJ	52.50±2.41g	63.34±2.18de	72.03±2.45b	43.23±2.17f
CJ	69.75±2.83bcd	64.33±0.95cde	41.43±2.39g	76.15±1.80b
JQWC	41.10±2.60h	68.67±0.31cd	54.40±3.97e	64.16±3.39de
CCSJ	44.24±2.71h	86.47±2.22a	49.93±2.25ef	74.26±2.57bc
BYH	86.72±2.59a	47.43±1.95gh	76.15±1.58b	70.29±1.92bcd
WCDHP	69.93±1.42bc	51.14±1.28fg	42.80±2.90fg	87.88±1.09a

HGHJ	52.59±2.18g	84.57±3.20a	85.09±3.97a	71.29±1.49bc
JYH	53.51±4.08fg	76.62±1.66b	63.91±0.82c	82.92±1.22a
Average value	62.58±0.43de	63.66±0.25de	62.57±0.75cd	72.66±0.41bc
F value	78.77**	151.13**	117.08**	142.73**
